# Cell Adhesion-Related Molecules Play a Key Role in Renal Cancer Progression by Multinetwork Analysis

**DOI:** 10.1155/2019/2325765

**Published:** 2019-12-16

**Authors:** Anbang Wang, Ming Chen, Hui Wang, Jinming Huang, Yi Bao, Xinxin Gan, Bing Liu, Xin Lu, Linhui Wang

**Affiliations:** ^1^Department of Urology, Changzheng Hospital, Second Military Medical University, Shanghai 200003, China; ^2^Department of Urology, Changhai Hospital, Second Military Medical University, Shanghai 200433, China

## Abstract

Renal cell carcinoma (RCC) is one of the most common malignancies in the urinary system. The study aimed to identify genetic characteristics and reveal the underlying mechanisms in RCC. GSE53757, GSE46699, and TCGA KIRC database (*n* = 897) were analyzed to screen differentially expressed genes (DEGs) in RCC. The gene ontology (GO) and Kyoto Encyclopedia of Genes and Genomes (KEGG) pathway enrichment analyses were performed, followed by the analysis of the protein-protein interaction (PPI) network of the DEGs by Cytoscape software. In all, 834 DEGs were identified in RCC, including 416 upregulated genes and 418 downregulated genes. The top 10 hub genes, VEGFA, EGFR, EGF, CD44, CD86, FN1, ITGAM, ITGB2, TLR2, and PTPRC, were identified from the PPI network according to the core degree. The following subnetwork revealed that these significant modules were enriched in positive regulation of response to external stimulus, regulation of leukocyte-mediated immunity, and regulation of exocytosis. The expressions of these hub genes were also validated using qRT-PCR and IHC in Changzheng RCC database (*n* = 160). We especially found that half of the top ten hub genes were cell adhesion-related molecules, which were associated with RCC progression and poor prognosis. In conclusion, these hub genes, particularly cell adhesion-related molecules, could be used as prognostic biomarkers and potential therapeutic targets for RCC.

## 1. Introduction

Renal cell carcinoma (RCC) is the third-leading cause of death among urological tumors, and the estimated numbers of new RCC cases and deaths are 62,700 and 14,240, respectively, in the USA for 2016 [1]. The incidence of RCC has been rising for over two decades [2]. The localized RCC and metastatic RCC fairly differ in consideration of prognosis and therapeutic approach. The 5-year cancer-specific survival is more than 70% in nonmetastatic RCC but dramatically decreases to less than 27.1% in metastatic RCC [3]. Compared with other cancers, there are very few tumor markers for RCC [4]. Thus, reliable molecular markers are urgently needed for the early prognosis of RCC. Like other cancers, RCC is considered as a heterogeneous disease in which gene aberrations, cellular context, and environmental influences synergistically lead to tumor initiation, progression, and metastasis [5]. Numerous studies have shown that multiple genes and cellular pathways are involved in the occurrence and development of RCC [6, 7]. However, the precise molecular mechanisms underlying RCC progression remain ambiguous, limiting the ability to treat advanced disease. Therefore, it is necessary to understand the molecular mechanism involved in proliferation, apoptosis, and invasion of RCC for the development of more effective diagnostic and therapeutic strategies.

Gene expression analyses based on microarray technology are promising tools in medical oncology, which can simultaneously detect changes in the expression of thousands of genes at the mRNA level. These high-throughput platforms bring great clinical applications, including molecular diagnosis, prognosis prediction, and new drug target discovery [8]. By gene expression profiling analysis on RCC, some research has found many differentially expressed genes (DEGs) involved in crucial signaling pathways or biological processes. However, there is no reliable biomarker profile to discriminate RCC from normal tissue, at least in part, because some results are defective or inconsistent due to limited sample and tissue heterogeneity [9]. Several studies have identified some key genes and signaling pathways in kidney cancer by biological information analysis [10, 11]. They identified some key genes, but there were some limitations, such as the small sample size and the lack of a comprehensive analysis of the relationship between these genes and the patient's clinical characteristics. The integrated bioinformatics combined with expression profiling analysis with large-scale samples might solve the problems. Copland et al. analyzed the DEGs using 72 pairs of renal cancer tissues and adjacent samples [12]. They indicated neuronal pentaglobin 2 as a new molecular target for the treatment of RCC patients diagnosed with metastatic disease or at risk of metastatic disease. Eckel-Passow et al. also found two key cancer-promoting genes, ANKS1B and ENRAGE, using 130 renal cancer and adjacent tissue expression profiling arrays [13]. However, the interactions among DEGs, particularly the complicated signaling pathways in the interaction network, remain to be elucidated.

In the current study, we used a systematic approach that may acquire novel molecular biomarkers for renal cancer. Based on the original data from GEO online database, we identified the differentially expressed genes (DEGs) and key molecular pathways and thus constructed a regulatory network. Then, the top 10 hub genes were selected and determined by RT-qPCR, IHC assay, and survival analysis. These hub genes could be used as potential biomarkers and therapeutic targets. Furthermore, they may bring a novel insight into renal cancer pathogenesis.

## 2. Materials and Methods

### 2.1. Acquisition of Microarray Data and Screening of DEGs

Gene Expression Omnibus (GEO, http://www.ncbi.nlm.nih.gov/geo/) database is a public functional genomics data repository, which store curated gene expression datasets, original series, and platform records. RCC-associated datasets GSE53757 and GSE46699 were downloaded from the GEO database. GSE53757, which was based on GPL570 platform, was submitted by Von Roemeling et al. The GSE53757 dataset contained 144 samples, including 72 RCC tissues across all stages of disease and matched normal kidney tissues [12]. The GSE46699 dataset analyzed expression profiling arrays of 130 renal cancer and adjacent tissues [13].

### 2.2. Screening of DEGs

The raw data files of GSE53757 and GSE46699 included TXT format data, which were processed using GEO2R (http://ncbi.nlm.nih.gov/geo/) under the R environment. GEO2R is used for online retrieval and analysis of biological and medical data. We also analyzed the DEGs of TCGA KIRC database using GEPIA website (http://gepia.cancer-pku.cn/). The statistically significant DEGs were defined with *p* < 0.05 and [log_2_ FC] ≥ 1.

### 2.3. Gene Ontology and Pathway Enrichment Analysis of DEGs

The gene ontology (GO) project is a useful method for consistently describing gene products across databases. GO terminology enrichment analysis includes biological processes, cellular components, and molecular functions [14, 15]. Kyoto Encyclopedia of Genes and Genomes (KEGG) pathway is a database resource for understanding the advanced functions and utilities of biological systems, especially large-scale molecular datasets generated by genome sequencing (https://www.kegg.jp/) [16]. In order to analyze biological roles of DEGs, GO and pathway enrichment analyses were performed using the Database for Annotation, Visualization, and Integrated Discovery (DAVID) online tool (https://david.ncifcrf.gov/). *p* < 0.05 was considered statistically significant.

### 2.4. Integration of Protein-Protein Interaction (PPI) Network and Module Analysis

To evaluate the interactive relationships among DEGs, PPI information of DEGs was retrieved from STRING. Subsequently, PPI was visualized by Cytoscape software (http://cytoscape.org/). The module analysis of PPI network was performed using the plug-in Molecular Complex Detection (MCODE). The hub gene analysis was identified by CytoHubba in Cytoscape. Besides, the pathway enrichment analyses were carried out for DEGs in the modules by Metascape.

### 2.5. Patients and Follow-Up

A total of 165 paired renal cancer and corresponding paracancerous tissues were obtained sequentially from patients undergoing radical or partial nephrectomy from 2008 to 2010 in Changzheng Hospital. This study was approved by the ethics committee of the Changzheng Hospital of Second Military Medical University, and all patients provided written informed consent. 165 paired renal cancer and paracancerous tissues in Changzheng RCC database were used to construct tissue microarrays (TMAs) by Wuhan Baiaosi Bioscience. 160 of the 165 patients have comprehensive information of clinicopathological traits and survival for complete analysis (supplementary [Supplementary-material supplementary-material-1]).

### 2.6. RNA Isolation and qRT-PCR Analysis

Total RNA was extracted by TRIzol (Invitrogen, USA). Real-time quantitative PCR was performed on triplicate samples in a reaction mix of SYBR Green (Takara, China) by ABI 7900HT Fast Real-Time PCR System (Applied Biosystems, USA). The expression of indicated genes was normalized to endogenous reference control *β*-actin by using 2-ΔΔCt method. Sequences of primers used for qRT-PCR in this study are listed in supplementary [Supplementary-material supplementary-material-1]. The mRNA expression of hub genes was determined using Changzheng RCC database (*n* = 40).

### 2.7. Immunohistochemistry and Survival Analysis

Paraffin-embedded sections of TMAs were deparaffinized and rehydrated, followed by tissue antigen repair. The antibodies against CD44, CD86, FN1, ITGAM, and ITGB2 were obtained from Abcam. Slides were then treated with polyperoxidase-conjugated IgG (OriGene). Staining was performed under a microscope with diaminobenzidine solution (Dako, USA) and counterstained with hematoxylin (Sigma Chemical Co., USA). The survival analyses of the top ten hub genes were performed using KIRC database (*n* = 530) in Kaplan–Meier plotter [17]. The protein expression of cell adhesion-related molecules in renal cancer and normal tissues was determined by IHC (*n* = 160). We also analyzed the relationship of cell adhesion-related molecules' expression with RCC clinicopathological traits and survival outcome.

### 2.8. Statistical Analysis

All statistical analyses in this study were performed with SPSS 18.0 software (SPSS Inc., USA). Data were presented as “mean ± SD.” The significance of mean values between two groups was analyzed by two-tailed Student's *t*-test. A *p* value <0.05 was considered to represent a statistically significant result.

## 3. Results

### 3.1. The Identification of DEGs in RCC

**Figure 1:**
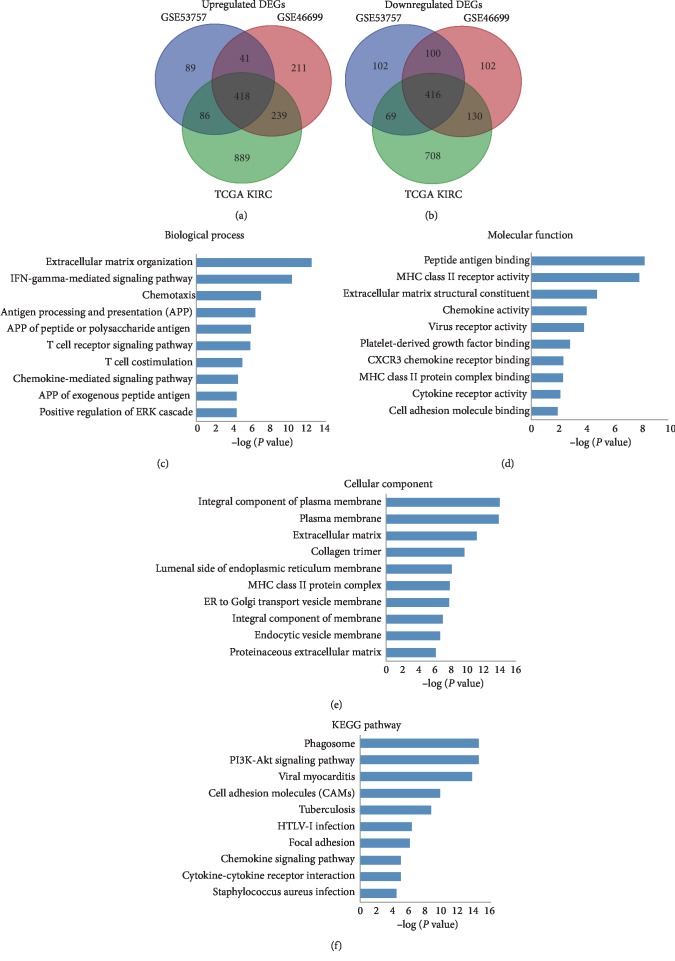
lncRNAs in a large database analysis comparing RCC samples to paracancerous tissues (a-b). The results are shown in a Venn diagram. Those on the right were upregulated in the intersection of 3 datasets (a). The left presents those downregulated at the intersection of 3 datasets (b). Gene ontology (c–e) and KEGG pathway (f) analysis of the upregulated differentially expressed genes associated with renal cancer. The threshold was a fold change ≥2.0 and a *p* value <0.05. BP: biological process; MF: molecular function; CC: cellular component.

First, we investigated the differential gene expression between RCC tissues and normal tissues in two GEO datasets (GSE53757 and GSE46699) and the TCGA database (662 tumor tissues and 235 normal tissues). The threshold we used to screen upregulated or downregulated genes was a fold change ≥2.0 and a *p* value <0.05. From the intersection of the transcriptome sequencing data, 834 differentially expressed genes were initially obtained, including 416 upregulated and 418 downregulated DEGs (Figures 1(a) and 1(b)).

### 3.2. GO Term Enrichment and KEGG Pathway Analysis of DEGs

All DEGs were uploaded to the David website to determine typical GO term classification and KEGG pathways. The results indicated that upregulated DEGs were enriched in extracellular matrix organization, interferon-gamma-mediated signaling pathway, and chemotaxis in the biological process (BP) (Figure 1(c)). The downregulated DEGs were mainly enriched in sodium ion homeostasis, negative regulation of growth, and gluconeogenesis (supplementary [Supplementary-material supplementary-material-1]). As for molecular function (MF), the overexpressed DEGs were significantly enriched in peptide antigen binding, extracellular matrix structural constituent, and chemokine activity (Figure 1(d)), and the downregulated DEGs were significantly enriched in anion antiporter activity and oxidoreductase activity (supplementary [Supplementary-material supplementary-material-1]). In addition, GO cell component (CC) analysis also displayed that the upregulated DEGs were significantly enriched in integral component of plasma membrane and plasma membrane (Figure 1(e)), and downregulated DEGs were enriched in integral component of membrane and plasma membrane (supplementary [Supplementary-material supplementary-material-1]).

As shown in Figure 1(f), the upregulated DEGs were enriched in Phagosome and PI3K-Akt signaling pathway, while the downregulated DEGs were enriched in amino acid metabolism and mineral absorption (supplementary [Supplementary-material supplementary-material-1]). These analysis results were different from GO terms enrichment analysis, to some extent, indicating complicated potential molecular mechanism involving in RCC. These significantly enriched pathways and terms may help to further understand the role that DEGs played in RCC development and progress.

### 3.3. Module Screening from the PPI Network

**Figure 2:**
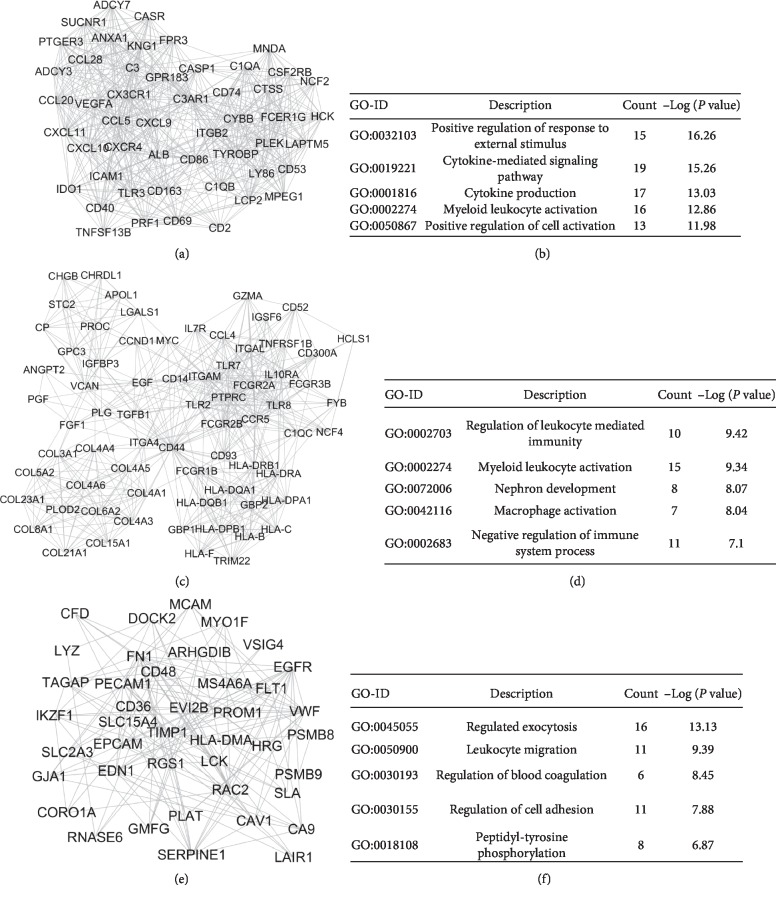
Module analysis from the protein–protein interaction network. The modules 1–3 consisted of 50, 70, and 41 nodes, respectively (a, c, e). The enrichment analysis of modules 1–3 (b, d, f).

Based on STRING database, protein-protein interaction (PPI) network of DEGs was constructed by Cytoscape software. The PPI network analysis also identified the top ten hub genes of RCC, which included protein tyrosine phosphatase, receptor type C (PTPRC), vascular endothelial growth factor A (VEGFA), epidermal growth factor receptor (EGFR), fibronectin 1 (FN1), CD44 molecule (CD44), integrin subunit alpha *M* (ITGAM), epidermal growth factor (EGF), CD86 molecule (CD86), integrin subunit beta 2 (ITGB2), and Toll-like receptor 2 (TLR2). Moreover, a total of 816 nodes and 6356 edges were analyzed using plug-in MCODE in Cytoscape software. The top 3 significant modules were obtained and were enriched in positive regulation of response to external stimulus, regulation of leukocyte mediated immunity, and regulation of exocytosis, respectively (Figure 2).

### 3.4. Validation of Hub Genes via RT-PCR, IHC, and Survival Analysis

**Figure 3:**
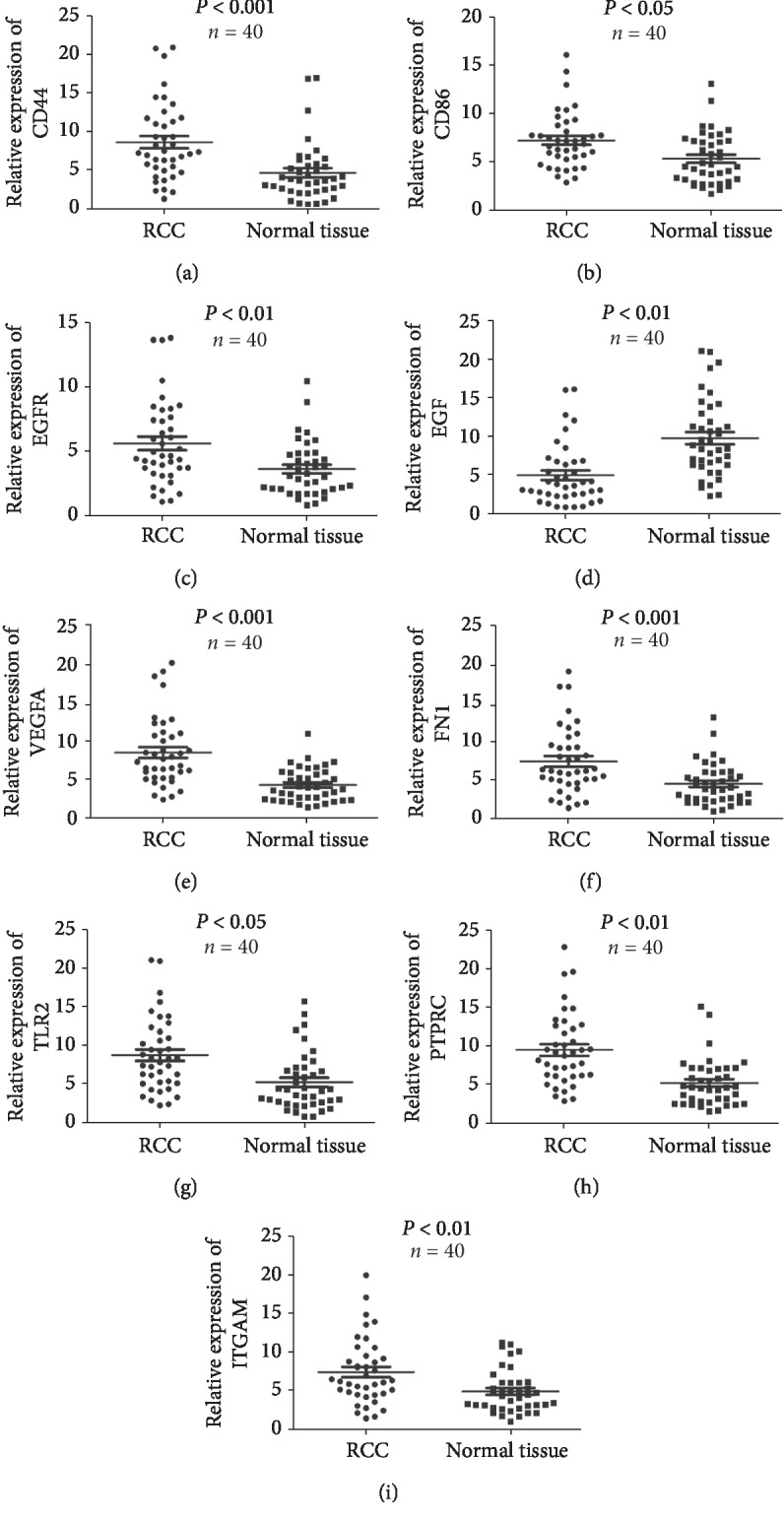
The top nine hub genes expression between RCC samples and paired normal tissues was compared using qRT-PCR analysis (*n* = 40). *p* < 0.05 by the Mann–Whitney *U* test.

**Figure 4:**
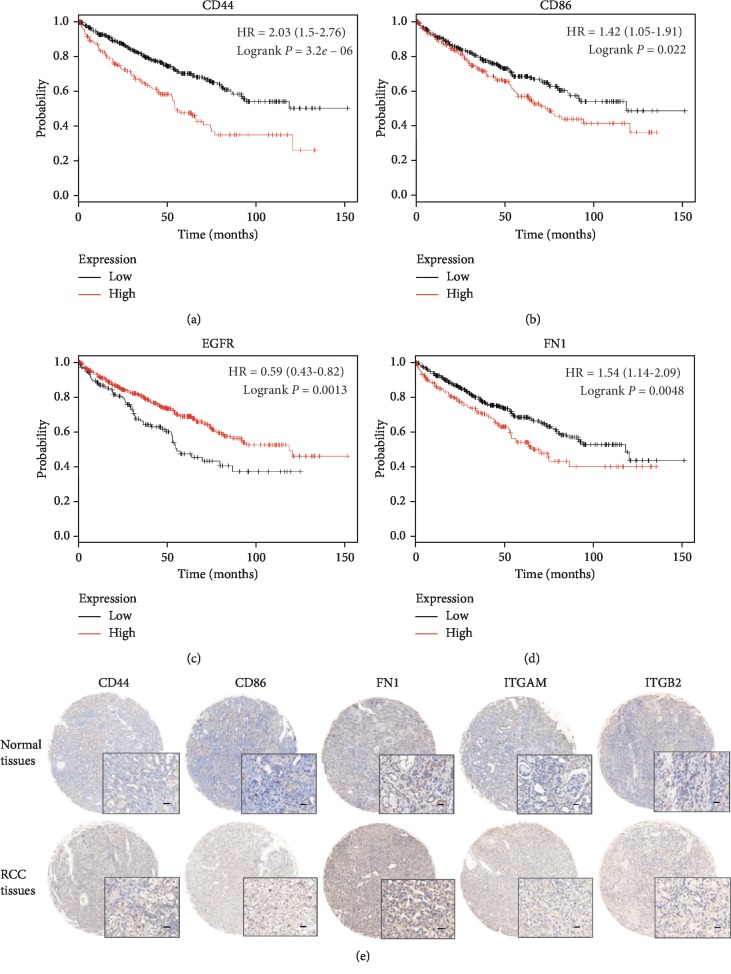
The survival analyses of hub genes, including CD44, CD86, EGFR, and FN1 (a–d). *p* < 0.05 was considered statistically significant. Representative immunohistochemistry images of cell adhesion-related molecules using tissue microarrays of human RCC and normal tissues (e) (*n* = 160). Black scale bar represents 50 *μ*m.

To confirm the key genes identified by the above analyses, we performed RT-PCR, IHC, and survival analysis of these hub genes by using Changzheng RCC database. Most of these hub genes, except EGF, were upregulated DEGs, which were validated in 40 paired renal cancer and paracancerous tissues of Changzheng RCC database (Figure 3, supplementary [Supplementary-material supplementary-material-1]). Survival analyses were performed using Kaplan–Meier plotter. The high expression of CD44, CD86, FN1, and TLR2 was correlated with a poor patient survival rate in RCC (Figure 4, supplementary [Supplementary-material supplementary-material-1]). The high expression of EGFR and EGF was correlated with a significantly longer overall survival of RCC patients (Figure 4, supplementary [Supplementary-material supplementary-material-1]). The high expression of ITGAM and ITGB2 indicated poor survival of RCC patients, although without sufficient statistical significance (supplementary [Supplementary-material supplementary-material-1]). Besides, although VEGFA expression appears to be unrelated to patient outcomes, our analysis indicated that VEGFA remains to be one hub gene of RCC. Currently, sunitinib based on VEGFA/VEGFR target is the first line of treatment for advanced renal cancer.

### 3.5. The Key Role of Cell Adhesion-Related Molecules

We noticed that half of the top ten hub genes were cell adhesion-related molecules, including FN1, ITGAM, ITGB2, CD44, and CD86. These cell adhesion-related molecules were mainly enriched in plasma membrane and extracellular region (Figure 4(e)). These molecules were highly expressed in RCC tissues than normal tissues according to IHC score in tissue microarrays (Figure 4(e)). We also analyzed the correlation between cell adhesion-related molecules expression and clinicopathological traits in 160 cases of RCC patients (Changzheng cohort). The results suggested that FN1, ITGAM, ITGB2, CD44, and CD86 were expressed at substantially higher levels in tumors >4 cm than in tumors ≤4 cm, in Fuhrman III/IV grade tumors than in Fuhrman I/II grade tumors, and in the distant metastasis group than in the no metastasis group (Table 1). These results indicated that cell adhesion progress was involved in RCC progression.

In conclusion, our results indicated that these hub genes were significantly differentially expressed in renal cancer compared to adjacent tissues. In particular, cell adhesion and growth factor-related molecule play the pivotal role in RCC development and metastasis. These hub genes and related signaling pathways may be used as useful biological biomarkers and therapeutic targets for the treatment of RCC.

## 4. Discussion

RCC is a heterogeneous disease, in which gene mutation, intracellular component, and extracellular microenvironment synergistically influence tumor initiation and progression [5]. It is of critical importance to understand the potential mechanism underlying RCC. High-throughput sequencing and microarray analysis have been widely used to find potential diagnostic and therapeutic targets for RCC. In the present study, we analyzed these high-throughput sequencing samples of RCC, whose samples consisted of 662 RCC tissues and 235 normal tissues. We identified 416 upregulated and 418 downregulated DEGs between RCC and normal control samples. In order to better understand the interactions of DEGs, we performed a series of bioinformatics analyses on DEGs to find RCC-associated genes and pathways.

The GO term enrichment analysis showed that upregulated DEGs were mainly involved in extracellular matrix organization, interferon-gamma-mediated signaling pathway, and chemotaxis, and downregulated DEGs were mainly enriched in sodium ion homeostasis, negative regulation of growth, and gluconeogenesis. Extracellular matrix plays a crucial role in the development of RCC. Coincidentally, we also found multiple adhesion-related molecules in the hub genes. Cancer immune evasion is a major stumbling block in designing effective anticancer therapeutic strategies [18]. The strategy to interrupt immune checkpoints such as anti-PD-1 may unleash antitumor immunity and mediate durable cancer regressions. A recent clinical trial reported that nivolumab plus ipilimumab showed higher overall survival and objective response rates than sunitinib in advanced RCC patients [19]. Besides, ion transport in cancer cells differs substantially from normal cells [20].

The KEGG pathway analysis indicated that upregulated DEGs were enriched in phagosome and PI3K-Akt signaling pathway, and downregulated DEGs were mainly involved in metabolic pathways. The cytokine VEGF/VEGFR pathway has been developed to be targeted by the small-molecule inhibitors in RCC. Other cytokines, such as TGF-*β*, EGFR, and CXCR4, have been reported in promoting RCC progression, metastasis, and survival. The PI3K-AKT pathway is overactivated in many human cancers, and several drugs that inhibit this pathway, including the PI3K/mTOR dual inhibitor NVP-BEZ235, are currently being tested in various preclinical and clinical trials. In addition, the PI3K-AKT pathway is an effective therapeutic target in RCC [21]. RCC is a metabolic disease characterized by dysregulation of metabolic pathways involved in oxygen sensing (changes in the VHL/HIF pathway and subsequent upregulation of HIF response genes such as VEGF, PDGF, and EGF), energy sensing (succinate dehydrogenase-deficient RCC), and/or nutrient-sensing cascades (dysregulation of the AMPK-mTOR and PI3K-AKT pathways). [22]. Their view was consistent with our studies. Therefore, those altered signaling pathways may represent potential targets for developing more effective therapeutic strategies.

The PPI network analysis of DEGs showed the top ten hub genes: VEGFA, EGFR, EGF, CD44, CD86, FN1, ITGAM, ITGB2, TLR2, and PTPRC. VEGFA, encoding vascular endothelial growth factor A, is observably related to the initiation and progression of cancer. Several targeted inhibitors targeting VEGF/VEGFR pathway have been widely used for the treatment of advanced RCC patients [23]. Epidermal growth factor receptor (EGFR) belongs to the receptor tyrosine kinase of the ErbB family. Our study found that EGFR-AS1 enhances the malignant phenotype of RCC cells by enhancing HuR mediated mRNA stability of EGFR [24]. EGFR was upregulated in RCC tissues and predicted poor prognosis of RCC patients. PTPRC and TLR2 were both significantly upregulated in clear cell RCC compared to normal renal samples [25]. However, their potential mechanisms were still not clear in RCC. The other hub genes were cell adhesion-related molecules. The CD44 antigen is a cell-surface glycoprotein involved in cellular interactions, cell adhesion, and migration. The expression of CD44 is associated with cancer cell invasion, metastasis, sunitinib resistance, and poor prognosis of RCC [26]. Schütz et al. demonstrated that CML patients with high CD86^+^ pDC counts have a higher risk of relapse after TKI discontinuation [27]. Han et al. indicated that ER*β*/circATP2B1/miR-204-3p/FN1 axis promotes invasion of clear cell RCC and may be potential prognostic biomarkers for this disease [28]. ITGAM and ITGB2 are subunits of integrin, which is correlated with poor prognosis of RCC patients [29]. These cell adhesion-related molecules are closely related to cancer cell invasion and metastasis. Further studies are needed to determine their role in RCC.

Module analysis on the PPI network indicated that the development of RCC was associated with positive regulation of response to external stimulus, regulation of leukocyte mediated immunity, and regulated exocytosis. The ECM-receptor interaction and focal adhesion are integral in maintaining cellular physiology. These pathways regulate cellular biological processes, including survival, proliferation, and migration by mediating cell signal transduction. A recent study reported that many cellular adhesion and extracellular matrix molecules were dysregulated in RCC tissues and correlated with the survival of renal cancer patients [29]. PI3K/AKT/mTOR pathway, composed of different kinases, is hyperactivated in human clear cell RCC. A lot of protein mutations were found in that pathway and were responsible for the dysregulation of cell proliferation, invasion, angiogenesis, and survival. Notably, the mTOR inhibitor such as everolimus has been used as a second-line treatment in advanced renal cancer. Exosomes are increasingly attracting attention as an important form of cellular exocytosis. Our study found that RCC cells can disseminate sunitinib resistance to sensitive cells through exosomes carrying lncARSR [30].

## 5. Conclusion

In conclusion, we identified a number of key genes and pathways associated with RCC initiation and progression through a comprehensive bioinformatics analysis of DEG. The following series of analyses may help to improve our understanding of the etiology and potential molecular events of RCC. Our analysis provides some candidate genes and pathways for future study that may serve as useful biomarkers and therapeutic targets. However, further researches are warranted to explore the function of the identified genes and pathways in RCC.

## Figures and Tables

**Table 1:** Correlations between cell adhesion-related molecule expression and clinicopathological features (*n* = 160).

Variables	FN1	ITGAM	ITGB2	CD44	CD86
Gender	0.665	0.389	0.483	0.118	0.069
Age	0.584	0.882	0.588	0.382	0.445
Tumor size, cm	0.017	0.008	0.015	0.012	0.083
Fuhrman grade	0.018	0.026	0.062	0.036	0.046
TNM stage	0.013	0.011	0.032	0.009	0.028
Distant metastasis	0.021	0.007	0.044	0.006	0.033

*p* values were determined according to high versus low expression of cell adhesion-related molecule. ^*∗*^*p* values <0.05 were considered statistically significant.

## Data Availability

The data used to support the findings of this study are included within the article.

## Supplementary Materials

Supplementary Materials Table S1: clinical characteristics of RCC patients. Table S2: sequences of primers used for qRT-PCR in this study. Figure S1: gene ontology (a–c) and KEGG pathway (d) analysis of the downregulated differentially expressed genes associated with renal cancer. Figure S2: (a) the ITGB2 mRNA expression between RCC samples and paired normal tissues was compared using qRT-PCR analysis (*n* = 40). (b) The survival analyses of hub genes, including EGF, VEGFA, TLR2, PTPRC, ITGAM, and ITGB2.
