# Supplementary material for: Cell Adhesion-Related Molecules Play a Key Role in Renal Cancer Progression by Multinetwork Analysis
Source: Biomed Res Int. 2019 Dec 16;2019:2325765. doi: 10.1155/2019/2325765 (PMC6948336; doi:10.1155/2019/2325765)

Supplementary Table

Table S1. Clinical characteristics of RCC patients

Variables	RCC patients (n=160)
**Gender (%)**	
Male	108 (67.5)
Female	52 (32.5)
**Age (%)**	
≤60	94 (58.8)
＞60	66 (41.2)
**Tumor size (%)**	
≤4cm	85 (53.1)
＞4cm	75(46.9)
**Fuhrman grade (%)**	
Ⅰ/Ⅱ	123 (76.9)
Ⅲ/Ⅵ	37 (23.1)
**TNM stage (%)**	
Ⅰ/Ⅱ	126 (78.8)
Ⅲ/Ⅵ	44 (21.2)
**Tumor thrombus (%)**	
No	148 (92.5)
Yes	12 (7.5)
**Distant metastasis (%)**	
No	144 (90.0)
Yes	16(10.0)

Table S2. Sequences of primers used for qRT-PCR in this study

CD44 Forward	GGATCACCGACAGCACAGAC
CD44 Reverse	TTCTTGCCTCTTGGTTGCTG
CD86 Forward	TAGGTCACAGCAGAAGCAGC
CD86 Reverse	AATCAAAACTTGTGCGGCCC
EGFR Forward	TGGTCAAGTGCTGGATGATAGA
EGFR Reverse	ACGGTAGAAGTTGGAGTCTGTA
EGF Forward	ACGCAGTTCCCACCCATTT
EGF Reverse	AACTCTGACTCTGAATGC
VEGFA Forward	AGGCCAGCACATAGGAGAGA
VEGFA Reverse	CTGGTTCCCGAAACCCTGAG
FN1 Forward	GACACATTCCACAAGCGTCA
FN1 Reverse	CTCCAATTTGATAAAACGTCCC
TLR2 Forward	CTCCCAGTGTTTGGTGTTGC
TLR2 Reverse	GGCTTGAACCAGGAAGACGA
ITGAM Forward	TCAGCCCCTCCCATAGCCA
ITGAM Reverse	GCTCCTACAGTCAGGTCTACCA
ITGB2 Forward	CCAGGGCAGACTGGTAGCAA
ITGB2 Reverse	AGAGGACTGAGGGACGAGGC

Supplementary Figure

Figure S1. Gene ontology (a-c) and KEGG pathway (d) analysis of the down-regulated differentially expressed genes associated with renal cancer. The threshold was a fold change ⩾ 2.0 and a p-value < 0.05. BP: biological process; MF: molecular function; CC: cellular component.

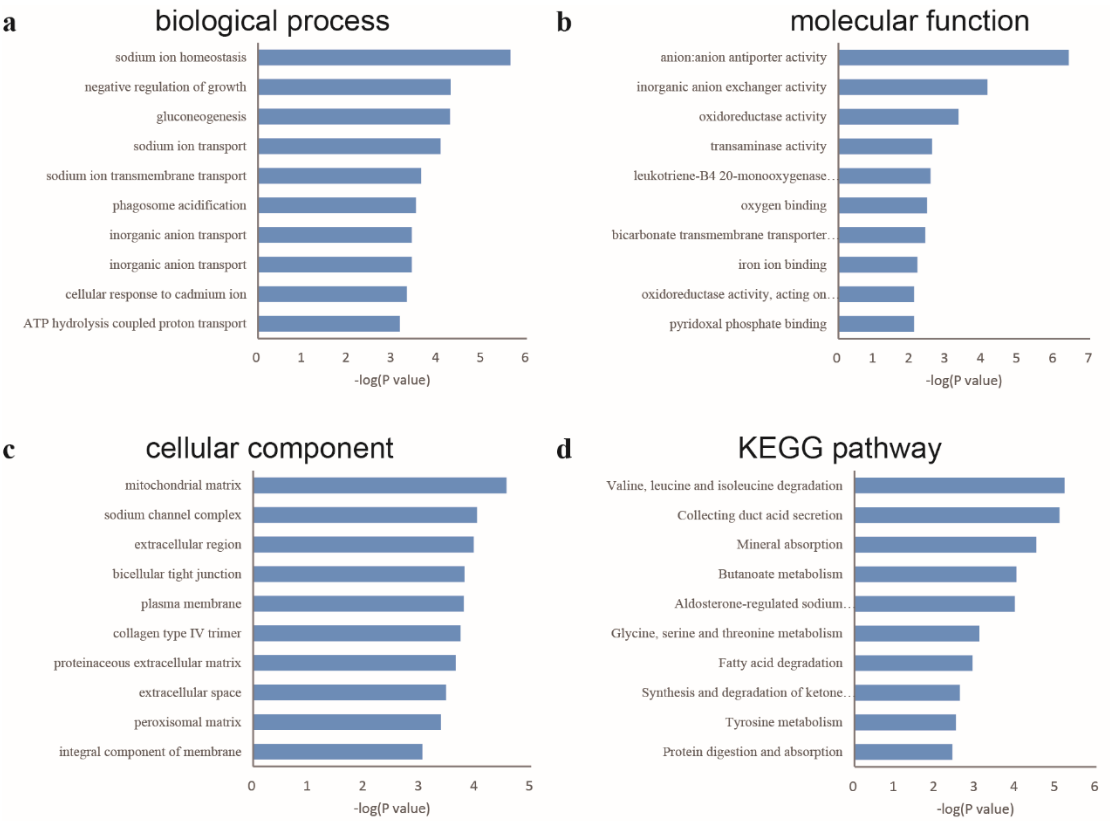

Figure S2. (a)The ITGB2 mRNA expression between RCC samples and paired normal tissues was compared using qRT-PCR analysis (n = 40). (b) The survival analyses of hub genes, including EGF, VEGFA, TLR2, PTPRC, ITGAM and ITGB2. p<0.05 was considered statistically significant.

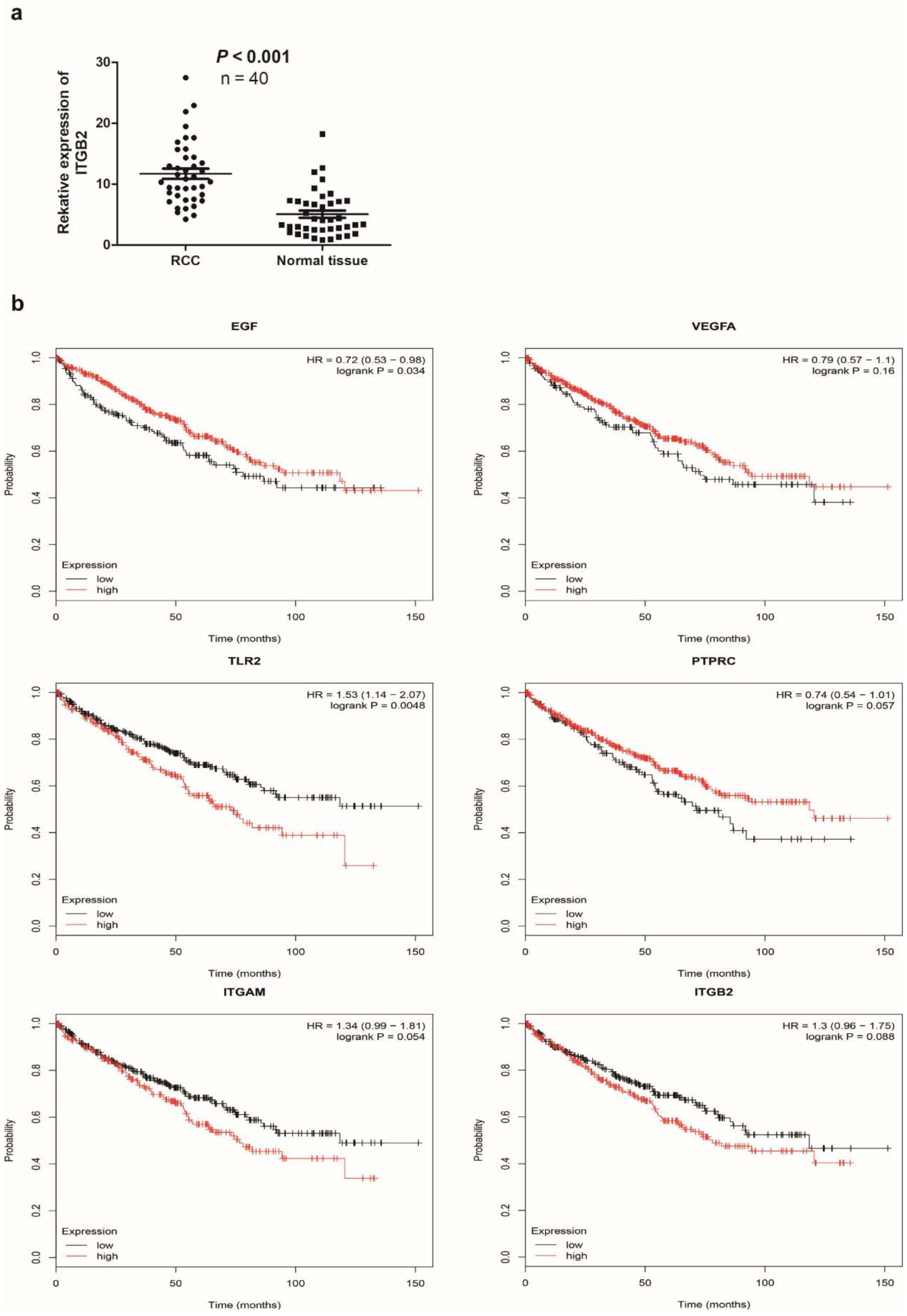

Supplement: Supplementary Materials — Table S1: clinical characteristics of RCC patients. Table S2: sequences of primers used for qRT-PCR in this study. Figure S1: gene ontology (a–c) and KEGG pathway (d) analysis of the downregulated differentially expressed genes associated with renal cancer. Figure S2: (a) the ITGB2 mRNA expression between RCC samples and paired normal tissues was compared using qRT-PCR analysis (n = 40). (b) The survival analyses of hub genes, including EGF, VEGFA, TLR2, PTPRC, ITGAM, and ITGB2. [file 2325765.f1.pdf]
